# N-Octanoyl Dopamine Inhibits the Expression of a Subset of κB Regulated Genes: Potential Role of p65 Ser276 Phosphorylation

**DOI:** 10.1371/journal.pone.0073122

**Published:** 2013-09-02

**Authors:** Maximilia C. Hottenrott, Johannes Wedel, Sophie Gaertner, Eleni Stamellou, Tineke Kraaij, Linda Mandel, Ralf Loesel, Carsten Sticht, Simone Hoeger, Lamia Ait-Hsiko, Angelika Schedel, Mathias Hafner, Benito Yard, Charalambos Tsagogiorgas

**Affiliations:** 1 Department of Anaesthesiology and Intensive Care Medicine, University Medical Centre Mannheim, Medical Faculty Mannheim, Ruprecht Karls University Heidelberg, Mannheim, Germany; 2 Vth. Medical Department, University Medical Centre Mannheim, Medical Faculty Mannheim, Ruprecht Karls University Heidelberg, Mannheim, Germany; 3 Department of Applied Chemistry, George-Simon-Ohm Hochschule, Nuernberg, Germany; 4 Centre for Medical Research (ZMF), Medical Faculty Mannheim, Ruprecht Karls University Heidelberg, Mannheim, Germany; 5 Institute of Transfusion Medicine and Immunology, Medical Faculty Mannheim, Ruprecht Karls University Heidelberg, Mannheim, Germany; 6 Institute for Molecular and Cellular Biology, Mannheim University of Applied Sciences, Mannheim, Germany; University of Oslo, Norway

## Abstract

**Background and Purpose:**

Catechol containing compounds have anti-inflammatory properties, yet for catecholamines these properties are modest. Since we have previously demonstrated that the synthetic dopamine derivative N-octanoyl dopamine (NOD) has superior anti-inflammatory properties compared to dopamine, we tested NOD in more detail and sought to elucidate the molecular entities and underlying mechanism by which NOD down-regulates inflammation.

**Experimental Approach:**

Genome wide gene expression profiling of human umbilical vein endothelial cells (HUVECs) was performed after stimulation with TNF-α or in the combination with NOD. Confirmation of these differences, NFκB activation and the molecular entities that were required for the anti-inflammatory properties were assessed in subsequent experiments.

**Key Results:**

Down regulation of inflammatory genes by NOD occurred predominantly for κB regulated genes, however not all κB regulated genes were affected. These findings were explained by inhibition of RelA phosphorylation at Ser276. Leukocyte adherence to TNF-α stimulated HUVECs was inhibited by NOD and was reflected by a diminished expression of adhesion molecules on HUVECs. NOD induced HO-1 expression, but this was not required for inhibition of NFκB. The anti-inflammatory effect of NOD seems to involve the redox active catechol structure, although the redox active para-dihydroxy benzene containing compounds also displayed anti-inflammatory effects, provided that they were sufficiently hydrophobic.

**Conclusions and Implications:**

The present study highlighted important mechanisms and molecular entities by which dihydroxy benzene compounds exert their potential anti-inflammatory action. Since NOD does not have hemodynamic properties, NOD seems to be a promising candidate drug for the treatment of inflammatory diseases.

## Introduction

Regulated transmigration of leukocytes across the endothelial lining of the vasculature is critical to both innate and acquired immunity. Inappropriate or excessive transendothelial migration is however undesired and can initiate many pathological processes. Temporal spatial regulation of the inflammatory response is therefore of utmost importance to prevent excessive inflammation in organs, and yet, to function adequately in combating infections. The inflammatory response is tightly regulated by mediators that activate the endothelium to express cell-associated adhesion molecules. Leukocyte transmigration starts with P- and E-selectin mediated transient binding to and rolling along the endothelium. Upon cytokine or chemokine activation, leukocytes firmly adhere to the endothelium [1] and subsequently leave the bloodstream using either of the two fundamentally different pathways, i.e. the para-cellular route requiring the opening of cell contacts [2] or the trans-cellular route through the body of endothelial cells [3]–[5].

The transcription factor NFκB is a family of closely related protein dimers that regulate inducible gene expression of pro-inflammatory mediators [6]. This family consists of five related proteins, i.e. p65 (RelA), RelB, c-Rel, p50/p105 (NFκB1) and p52 (NFκB2), which bind as dimer to common κB sequence motifs in promoters or enhancers of target genes. Subsequently, transcription is regulated through the recruitment of transcriptional co-activators and transcriptional co-repressors [7]. Inhibition of NFκB effectively down-regulates inflammation as has been shown in a number of experimental studies [8].

Plant-derived polyphenols are increasingly receiving attention as potential drugs for the treatment of a variety of pathological conditions [9]–[11]. Their beneficial effect seems to be directly related to structural entities within these compounds as reflected by differences in efficacy amongst individual polyphenols [12], [13]. The ability of para- and ortho-hydroquinone moieties within polyphenols to activate the Keap-1/Nrf-2/ARE pathway underscores the relevance of these entities for displaying cyto-protective properties [14]. While it has been unambiguously demonstrated that a number of polyphenols possess strong anti-inflammatory action, the underlying mechanism has been equivocally discussed in recent years. Although tested in different cells or cell lines obtained from different species, Nrf-2 mediated induction of HO-1 [15], [16], inhibition of NFκB [16], [17] and inhibition of PLA2 [13] all seems to be pivotal or contributing to the anti-inflammatory action.

In addition to polyphenols, there is also a huge body of evidence indicating that catecholamines have the propensity to modulate immune function in a pleiotypic manner affecting a variety of immune cells including monocytes, lymphocytes and NK (natural killer) cells [18], [19]. Modulation of the cytokine network by catecholamines occurs at (patho)-physiological concentrations and is mediated via engagement of adrenergic receptors [19]. Like polyphenols, catecholamines have the propensity to induce HO-1 [20], [21] and to inhibit the expression of inflammatory mediators in cultured endothelial and renal epithelial cells in a receptor independent fashion [22], [23]. Yet *in vitro*, their effective concentration to exert these anti-inflammatory properties by far surpasses clinical relevant concentrations, making as to whether catecholamines exert these anti-inflammatory properties *in vivo* questionable. Nonetheless, it should be emphasized that dopamine treatment in brain dead rats [24] or in rats subjected to renal ischemia [25] is associated with a reduction of inflammation, albeit that the mechanisms by which this occurs may largely differ from the *in vitro* findings.

We have recently synthesized a more hydrophobic dopamine derivative, i.e. N-octanoyl dopamine (NOD), which compared to dopamine displayed improved cellular uptake and does not elevate mean arterial blood pressure [26]. *In vitro*, NOD is approximately 50 times more effective than dopamine in protecting endothelial cells against hypothermic cell injury [26]. Moreover, not only the anti-inflammatory action of NOD is superior to that of dopamine, it is also more effective in reducing ischemia induced acute kidney injury in rats [27]. In the present study we investigated the anti-inflammatory properties of NOD in more detail. By making use of genome wide gene expression profiling, functional studies and structural variants of dihydroxy benzene derivatives we sought to elucidate the underlying molecular mechanism and molecular entities by which NOD down-regulates TNF-α mediated inflammatory responses.

## Materials and Methods

### Ethics Statement

Human umbilical vein endothelial cells (HUVECs) were received in collaboration with the Institute of Transfusion Medicine and Immunology, Medical Faculty Mannheim, Heidelberg University. Permission for isolation and propagation of endothelial cell from umbilical cords for research purposes was granted by the local ethic committee of the Clinical Faculty Mannheim, University of Heidelberg with informed consent in writing.

### Cell Culture

The HUVECs were grown in basal endothelial medium supplemented with 10% FBS and essential growth factors (Promo Cell, Heidelberg, Germany). Only cells in passage 4–6 were used in all experiments.

### Gene Expression Profiling

Sample preparation and processing was performed according to the Affymetrix GeneChip Expression Analysis Manual (http://www.Affymetrix.com). Total RNA was isolated HUVECs using Trizol^®^-Reagent (Life Technologies, Inc., Rockville, MD, USA). DNase treatment was carried out, using RNase free DNase I (Ambion, Woodward, Austin, TX, USA). RNA concentration and quality were assessed by RNA 6000 nano assays on a Bioanalyser 2100 system (Agilent, Waldbronn, Germany). Five µg of RNA was converted into cDNA using T7-(dT)_24_ primers and the SuperScript Choice system for cDNA synthesis (Life Technologies, Inc., Rockville, MD, USA). Biotin-labelled cRNA was prepared by *in vitro* transcription using the BioArray high yield RNA transcript labelling kit (Enzo Diagnostics, Farmingdale, NY, USA). The resulting cRNA was purified, fragmented and hybridized to U133A gene chips (Affymetrix, Santa Clara, CA, USA). After hybridization the chips were stained with streptavidin–phycoerythrin (MoBiTec, Goettingen, Germany) and analysed on a GeneArray scanner (Hewlett Packard Corporation, Palo Alto, CA, USA). The Raw fluorescence intensity values were normalized applying quantile normalization.

### FACS Analysis

FACS analysis was performed as described previously [28], using FITC-conjugated monoclonal antibodies directed against ICAM-1 (BBIG-I1), VCAM-1 (BBIG-V3) or E-selectin (BBIG-E5) (all from R&D Systems, Wiesbaden-Nordenstadt, Germany). FACS analysis was performed on a FACScalibur (Becton Dickinson, Heidelberg, Germany) equipped with the CELLQuest software. The data were analyzed by Windows Multiple Document Interface (WinMDI) software (Version 2.8).

### Adhesion Assays

HUVECs were seeded either in collagen coated 24 well plates or in flow chambers (ibidi, Munich, Germany) at a concentration of 10^6^ cells per ml. For cell adhesion under *static* conditions, the plates were washed and incubated for 30 min with 1 ml of 10^6^ carboxyfluorescein succinimidyl ester (CSFE) (In Vitrogen, Darmstadt, Germany) labelled peripheral blood mononuclear cells (PBMCs). PBMCs were isolated using Ficoll gradient centrifugation. CSFE labelling was performed according to the manufacturer’s instructions. The plates were extensively washed with PBS and remaining cells lysed with distilled water. The fluorescence in cell lysates was measured on a Tecan Infinite 200 with the appropriate filters (Tecan Group, Männedorf, Switzerland). For cell adhesion under *flow* conditions (0.6 dyn/cm^2^), ibidi chambers were subsequently perfused for 10 min with normal cell culture medium, than perfused for 10 min with cell culture medium containing 10^6^ PBMCs/ml and finally perfused for 5 min with normal cell culture medium to remove non adherent cells. All conditions were performed in triplicate. Each individual chamber PBMCs was counted in five random non-coincident microscopic fields (phase contrast). Counting was performed by two investigators without prior knowledge of the experimental conditions.

### Electrophoretic Mobility Shift Assay (EMSA)

HUVECs were stimulated for different time periods with 10 ng/ml of TNF-α alone or in combination with 50 µM of NOD. In some experiments the cells were pre-treated for 2 hrs with cyclohexamide (CyHx) (5 µg/ml) before stimulation. In these experiments the cells were stimulated for 8 hrs in the continued presence or absence of CyHx. Nuclear extracts were prepared as previously described [29]. Protein concentrations were determined by Bradford assay. EMSA was performed essentially as previously published [30], [31]. Briefly, NFκB (5′-AGTTGAGGGGACTTTCCCAGGC-3′) double-stranded consensus oligonucleotide (Promega Corp., Madison, WI, USA) was end-labeled with γ-^32^P-ATP using T4-polynucleotide kinase, ethanol precipitated and finally dissolved in 20 µl of distilled water. One µl of ^32^P-labeled probe (∼30,000 cpm) and 15 µg of nuclear extracts were added to a binding reaction mixture containing: 10 mmol/l HEPES (pH 7.5), 0.5 mmol/l EDTA, 70 mmol/l KCl, 2 mmo/l DTT, 2% glycerol, 0.025% NP-40, 4% Ficoll, 0.1 mol/l PMSF, 1 mg/ml bovine serum albumin and 0.1 mg/ml poly di/dc and incubated for 30 min at room temperature. DNA–protein complexes were separated by electrophoresis through a 5% non-denaturing acrylamide: bis-acrylamide gel in 0.5 × Tris–borate/EDTA (TBE) for 3 h at 220 V. Gels were analyzed by autoradiography using an Amersham Hyperfilm ECL (GE Healthcare Bio-Sciences AB, Uppsala, Sweden). In each experiment, specificity of binding was demonstrated by preincubation of cold consensus (100x excess of unlabeled oligonucleotide) or mutated NFκB oligonucleotide to the nuclear extracts. In addition, supershifts were performed by adding p50, p52, p65, RelB and c-Rel antibodies (all Santa Cruz Biotechnology, Heidelberg, Germany) to the samples.

### Western Blotting

HUVECs were lysed in lysis buffer (10 mM Tris, 2% SDS, 0.5% beta-mercaptoethanol) (all from Sigma-Aldrich, St. Louis, MO). Protein concentrations were measured using Coomassie-Reagent (Pierce, Rockford, USA). Samples (20 µg protein extract) were heated to 95°C for 5 min, loaded and separated on 10–20% SDS-polyacrylamide gels followed by semi-dry blotted onto PVDF membranes (Roche, Mannheim, Germany). Staining of blots was performed by standard operating procedures using polyclonal anti-VCAM-1, anti-HO-1, anti-Nrf-2 antibodies (all Santa Cruz Biotechnology, Heidelberg, Germany). To confirm equal protein loading, membranes were re-probed with monoclonal anti-GAPDH antibody (Abcam, Cambridge, UK).

### Cell Transfection with siRNA

HUVECs were seeded in 6 well plates at a density of 0.5–2×10^5^ one day before transfection with HO-1 siRNA, Nrf-2 siRNA or control siRNA (Santa Cruz Biotechnology, Heidelberg, Germany). Transfection was performed according to the manufacturer’s instructions. Briefly, cells were incubated for 6 hrs in transfection medium supplemented with siRNA and transfection reagent. Hereafter, endothelial cell culture medium containing 20% FBS was added without removing the transfection solution and the cells were allowed to grow for additional 24 hrs. For each experiment the efficacy of siRNA was demonstrated by disappearance of the specific band in Western blot analysis.

### Synthesis of Dihydroxy Benzoic Acid Derivatives

Two grams 2,5-dihydroxybenzoic acid was suspended in 5 ml acetic anhydride under magnetic stirring. When two drops of sulphuric acid were added, the suspension turned clear and stirring was continued for one hour. Diluted hydrochloric acid (5 ml) was added and 30 min later the reaction mixture was poured into 200 ml ice water. The precipitated product was collected by vacuum filtration and dried under vacuum to yield 2,5-bisacetoxybenzoic acid, pure as judged by thin layer chromatography (TLC). Bisacetoxybenzoic acid was reacted with stoichiometric amounts of ethyl chloroformate to obtain the mixed anhydride which was used without purification. The anhydride was dissolved in dimethyl formamide and the respective amine added in equal stoichiometric quantity. After reacting overnight, the mixture was diluted with ethyl acetate and the organic phase was extracted subsequently with neutral phosphate buffer, brine, diluted sulphuric acid and again brine. Drying over MgSO_4_ and removal of the solvent under vacuum yielded the crude product, which were recrystallized from aqueous ethanol.

### Statistics

Differential gene expression was analysed based on loglinear mixed model ANOVA, using a commercial software package SAS JMP7 Genomics, version 3.1, from SAS (SAS Institute, Cary, NC, USA). A false positive rate of a = 0.05 with Holm correction was taken as the level of significance. Pathways belonging to various cell functions such as cell cycle or apoptosis were obtained from public external databases (KEGG, http://www.genome.jp/kegg/). A Fisher’s exact test was performed to detect the significantly regulated pathways.

Statistical analyses of cell adhesion assays under *static* and *flow conditions* were performed using SigmaPlot 11.0 (Systat Software GmbH, Erkrath, Germany). Data were compared with the Kruskal-Wallis signed-rank test and Dunńs post hoc test when required. Statistical significance was defined as *p*<0.05. Descriptive statistics are expressed as mean ± SD.

For westernblots optical density of bands of all blots were assessed using ImageJ 1.46 and Student’s t-test with previous testing of equality of variances by SigmaPlot 11.0 (Systat Software GmbH, Erkrath, Germany) was performed. If equality test failed, the Kruskal-Wallis-test was performed.

## Results

### Anti-inflammatory Potential of NOD

To investigate the anti-inflammatory potential of N-octanoyl dopamine (NOD), we screened by genome wide gene expression profiling in HUVECs for genes that were down regulated by NOD. To this end, three different primary cultures of HUVECs were stimulated with TNF-α alone or in combination with 100 µM NOD. Two major differences were observed when an arbitrary cut-off for a fold change of at least 2 was chosen. Firstly, the expression of a number of genes encoding chemokines or adhesion molecules was strongly down-regulated, and secondly, down-regulation in genes which are believed to be involved in the ubiquitin-proteasome system (UPS) was noted. Enlisted in table 1 are chemokines and adhesion molecules that were more than 2 fold down-regulated by NOD, when comparing TNF-α vs. TNF-α +100 µM NOD. Changes in chemokine expression were found for the CCL and CXCL family members, but also for fractalkine (CX3CL1). Similarly, the expression of three major adhesion molecules, i.e. VCAM-1, ICAM-1 and E-selectin, was significantly reduced in the presence of NOD (table 1). Changes in gene expression for genes belonging to the UPS included ubiquitin ligases (UBE2L6 and HERC6), ubiquitin like modifiers (ISG15 and UBD) and several proteasome subunits (PSME1, PSMB10, PSMB9 and PSMB8) (table 2). Although in affymetrix analysis some of the signalling molecules belonging to the NFκB pathway were slightly reduced by NOD (TNF-α vs. TNF-α+NOD fold change as log2: RelB: 0,73; NFKB1: 0,66; NFKBIA: 0,80 and IKBKE: 0,86), qPCR analysis revealed only a significant change for RelA, RelB and NFKBIE in independent experiments (data not shown). The expression of 95 genes was more than 2 fold up-regulated by TNF-α+NOD compared to TNF-α alone. With the exception of HO-1 (HMOX1: fold change (log2) 4,37; *p*-value: 1,9E-22), these differences were not further analysed. The complete dataset, including normalised and raw data, are available at the GEO repository http://www.ncbi.nlm.nih.gov/geo/query/acc.cgi?token=pjuvzqmawywairu&acc=GSE34059 with accession number (GSE34059). The influence of NOD on VCAM-1, ICAM-1, E-selectin and HO-1 was confirmed by Taqman PCR in independent experiments (data not shown).

**Table 1:** Down regulations of chemokines and adhesion molecules by NOD.

Gene	Fold change (log2)^a^	*p*-value^b^
*chemokines*		
CCL2	1,35	9,7E-07
CCL5	3,40	3,1E-11
CCL20	3,09	5,3E-08
CXCL1	3,07	2,0E-13
CXCL2	2,66	5,2E-09
CXCL3	3,26	7,8E-12
CXCL5	4,26	2,2E-33
CXCL6	4,05	1,3E-08
CXCL10	5,42	5,5E-21
CXCL11	5,47	1,8E-29
CX3CL1	4,28	4,5E-18
*Adhesion molecules*		
VCAM1	6,11	7,9E-21
ICAM1	2,47	1,6E-18
SELE	4,94	5,7E-24

a fold change values are expressed as Log2, TNF-α compared to TNF-α plus 100 µM NOD.

b *p*-values for the comparison TNF-α vs. TNF-α plus 100 µM NOD are given as log10.

**Table 2:** Down regulation of UPS associated genes by NOD.

Gene	Fold change (log2)^a^	*p*-value^b^
*Ubiquitin ligases*		
UBE2L6	2,04	5,8E-11
HERC6	2,48	2,1E-12
*Ubiquitin like*		
UBD	5,60	2,1E-22
ISG15	2,68	3,7E-15
*Proteasome*		
PSME1	1,55	6,8E-12
PSMB10	1,75	7,0E-12
PSMB8	1,96	3,1E-11
PSMB9	3,46	2,2E-10

a fold change values are expressed as Log2, TNF-α compared to TNF-α plus 100 µM NOD.

b *p*-values for the comparison TNF-α vs. TNF-α plus 100 µM NOD are given as log10.

### NOD Impairs PBMCs Adhesion to Endothelial Cells

**Figure 1:**
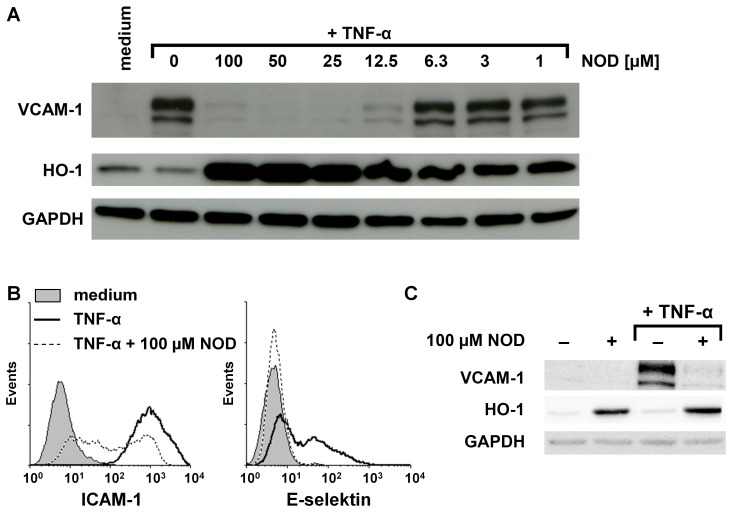
Influence of NOD on the expression of adhesion molecules and HO-1. (A) HUVECs were stimulated for 24 hrs with 10 ng/ml of TNF-α in the presence of different concentrations of NOD. The expression of VCAM-1 and HO-1 was assessed by western blotting. GAPDH was used as loading control. (B) HUVECs were stimulated as described in A. The expression of ICAM-1 and E-selectin was assessed by FACS analysis. (C) To demonstrate that the induction of HO-1 by NOD was independent of TNF-α, HUVECs were stimulated for 24 hrs with 10 ng/ml of TNF-α alone, 100 µM of NOD alone or in combination of both. HUVECs cultured in medium served as control. The expression of VCAM-1 and HO-1 was assessed by western blotting. The results shown in A, B and C are representative experiments. A total of 6 independent experiments with different HUVEC cultures were performed. All westernblots have been scanned and statistics was performed on the ratio of the optical density of protein of interest/optical density of GAPDH. Significant inhibition of VCAM-1 expression occurred at concentrations above 12.5 µM and HO-1 induction already at 1 µM (*p*<0.01).

Western blotting revealed that NOD dose-dependently inhibits TNF-α mediated VCAM-1 expression on protein level and confirmed that NOD induces the expression of HO-1 (figure 1). An almost complete inhibition of VCAM-1 was observed at a concentration of 12 µM of NOD, while induction of HO-1 was already noticed at 1 µM of NOD (figure 1A). Similar as demonstrated for VCAM-1 expression, FACS analysis revealed that TNF-α mediated up-regulation of E-selectin and ICAM-1 was blunted when the cells were stimulated with TNF-α in the presence of NOD (figure 1B). Induction of HO-1 expression was completely independent of TNF-α as HO-1 was also induced when cells were stimulated with NOD alone (figure 1C).

**Figure 2:**
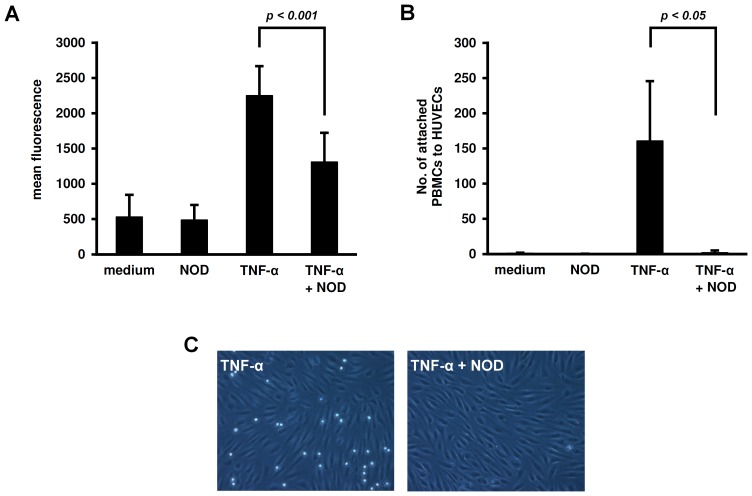
Influence of NOD on the adherence of PBMCs to endothelial cells. (A) Adherence of PBMCs was assessed under *static* conditions. To this end, HUVECs were seeded in 24 well plates and stimulated for 24 hrs with 10 ng/ml of TNF-α alone, 100 µM of NOD alone or with the combination of both. HUVECs cultured in medium served as control. CSFE labelled PBMCs were added to the plates for 30 min in a concentration of 10^6^ cells/well. Hereafter the plates were thoroughly washed and the fluorescence signal was measured in the cell lysates. All conditions were tested in triplicates and at least 4 independent experiments were performed. The results are expressed as mean fluorescence ± SD. (B) Adherence of PBMCs under *flow* conditions. HUVECs were seeded in ibidi flow chambers and stimulated as described in A. The chambers were flushed as described in the materials and methods section and adherent PBMCs were counted by two investigators without prior knowledge of the experimental conditions. All conditions were performed in triplicate and for each individual chamber five random microscopic fields (phase contrast) were counted. A total of 4 different experiments were performed the results are expressed as mean cell count ± SD. (C) A representative microscopic field is shown.

Under static conditions, adhesion of peripheral blood mononuclear cells (PBMCs) to HUVECs was significantly impaired when HUVECs were stimulated with the combination of TNF-α+NOD as compared to TNF-α alone (figure 2A). Also under flow conditions adhesion of PBMCs to HUVECs was strongly impaired when HUVECs were stimulated with TNF-α+NOD (figures 2B+C).

### NOD Inhibits Activation of NFκB

**Figure 3:**
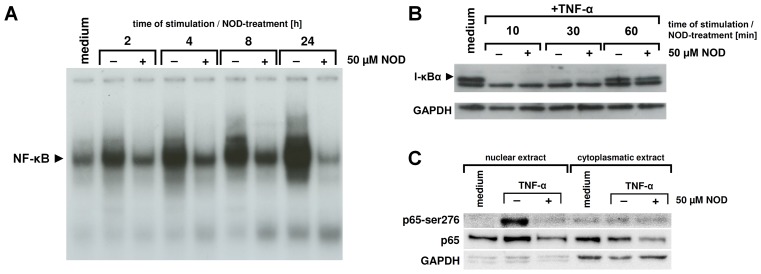
Influence of NOD on TNF-α mediated NFκB activation. (A) HUVECs were stimulated for different time periods with 10 ng/ml of TNF-α. During stimulation NOD (50 µM) was absent (−) or present (+). Nuclear extracts were prepared and assessed for NFκB activation by means of EMSA. (B) In separate experiments the influence of NOD on the degradation of IκBα was assessed. HUVECs were stimulated for different time periods in the presence (+) or absence (−) of 50 µM of NOD. HUVECs grown in medium served as control. IκBα degradation was assessed by western blotting. (C) HUVECs were stimulated for 24 hrs with 10 ng/ml of TNF-α (TNF-α) in the presence (+) or absence (−) of 50 µM of NOD. Cells that were left untreated (medium) served as control. Nuclear- and cytoplasmic extracts were prepared and assessed for the expression of phosphorylated p65 at Ser276 (p65- Ser276) and total p65 (p65) by western blotting. GAPDH was used as loading control. Note that in the condition of TNF-α+NOD phosphorylation of p65 at Ser276 is not detectable in the nuclear extracts and that expression of total p65 in both nuclear and cytoplasmic extracts is decreased. The results of a representative experiment are shown. A total of 4 independent experiments with different HUVEC cultures were performed.

TNF-α mediated expression of chemokines and adhesion molecules critically depends on activation of the NFκB transcription factor. We therefore tested if an impaired activation of NFκB could underlie the decreased mRNA expression of chemokines and adhesion molecules when NOD was present during TNF-α stimulation. NFκB was activated by TNF-α in a time dependent manner with maximal activation occurring at 24 hours of stimulation. Although in the presence of NOD activation of NFκB also occurred, it was at all studied time-points clearly diminished (figure 3A). Since Affymetrix analysis revealed that the expression of genes belonging to the UPS were significantly changed by NOD, we anticipated that NOD could potentially interfere with the degradation of IκBα. Ten minutes after TNF-α stimulation IκBα was nomore detected in western blot analyses but re-appeared after 30 min. The presence of NOD during stimulation did not influence degradation of IκBα (figure 3B). To exclude that the time of TNF-α stimulation would be too short for reaching sufficient intra-cellular concentrations of NOD, HUVECs were pre-treated for 24 hours with NOD and subsequently stimulated over a 1 hour-period with TNF-α in the continued presence of NOD. Degradation of IκBα still occurred in this experimental setting (data not shown), suggesting that NOD does not interfere with the initial events of NFκB activation.

Post-translational modifications of NFκB proteins are of eminent importance for transcriptional regulation of a number of NFκB regulated genes. In particular, phosphorylation of NFκB p65 on Ser276 seems to be instrumental for the recruitment of co-activators and subsequently gene transcription during inflammation [32], [33]. We therefore tested if Ser276 phosphorylation of NFκB p65 was affected by NOD and to what extent the expression of total NFκB p65 in nuclear and cytoplasmic extracts was influenced. In cells that were not stimulated, the expression of NFκB p65 was more prevalent in the cytoplasmic extract as compared to the nuclear extract. Upon stimulation with TNF-α, there was a shift towards a higher expression of NFκB p65 in the nuclear extract, compatible with its nuclear translocation. Interestingly, in cells that were stimulated with the combination of TNF-α+NOD the expression of NFκB p65 in both the cytoplasmic and nuclear extract was lower compared to cells that were not stimulated or stimulated with TNF-α alone (figure 3C, panel in the middle). Phosphorylation on Ser276 only occurred in the nucleus when the cells were stimulated with TNF-α. This was completely prevented when NOD was present during stimulation (figure 3C, upper panel).

### NOD Mediated Inhibition of NFκB does not Require HO-1

**Figure 4:**
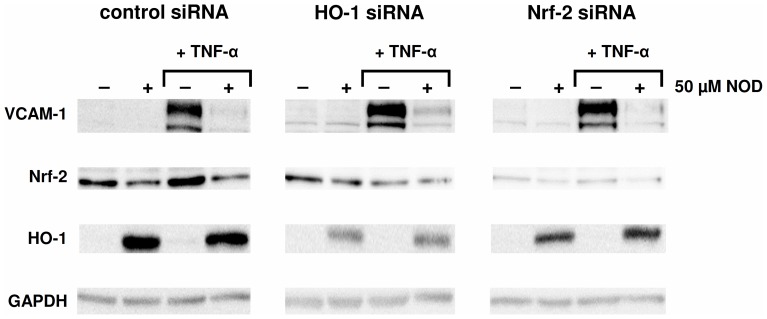
Partial silencing of Nrf-2 and HO-1 expression does not abrogate NOD mediated inhibition of VCAM-1. HUVECs were transfected with control siRNA, HO-1 siRNA or Nrf-2 siRNA. One day after transfection the cells were stimulated for 24 hrs with TNF-α alone (10 ng/ml), NOD alone (50 µM) or in combination of both. Cells that were not stimulated were included in each experiment. The expression of VCAM-1, Nrf-2 and HO-1 was assessed by western blotting. GAPDH was used as loading control. The results of a representative experiment are shown. A total of 3 independent experiments with different HUVEC cultures were performed.

**Figure 5:**
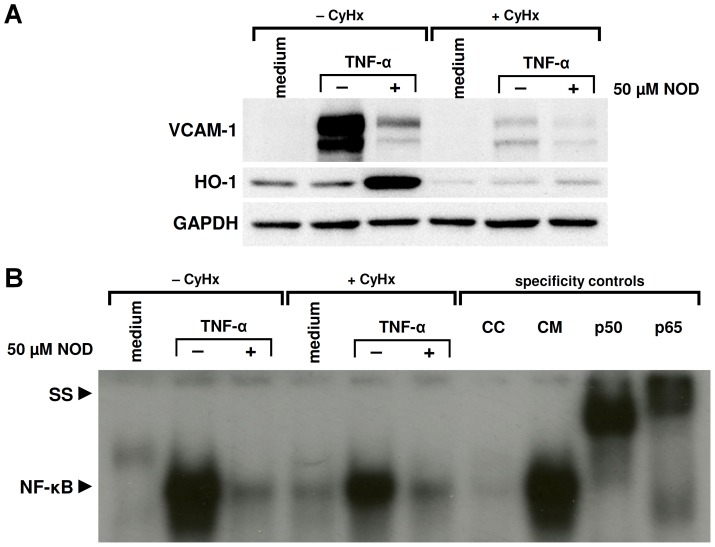
HO-1 induction by NOD is not required for inhibition of NFκB. (A) HUVECs were pre-treated for 2 hrs with 5 µg/ml of cyclohexamide (+ CyHx) or left untreated (− CyHx). Hereafter, cells were stimulated for 8 hrs with 10 ng/ml of TNF-α in the presence (+) or absence (−) of 50 µM of NOD. In case the cells were pre-treated with CyHx, this was present during the whole period of stimulation. In case cells were not treated with CyHx, this was absent during stimulation. Western blotting of the cytoplasmic fractions revealed that *de novo* protein synthesis was effectively inhibited by CyHx. Note that VCAM-1 is not induced by TNF-α in the presence of CyHx. Also in the combination of TNF-α+NOD the induction of HO-1 did not occur. GAPDH was used as loading control. (B) Nuclear extracts were prepared and assessed for NFκB activation by means of EMSA. Specificity of the bands was assessed by adding an excess of unlabelled NFκB consensus (cold consensus (CC)) or mutated (cold mutated (CM)) oligonucleotides to the samples. To demonstrate the presence of p50 and p65 in the shifted bands super-shifts (SS) were performed by adding anti-p50 or anti-p65 monoclonal antibodies to the samples. In A und B the results of a representative experiment are shown. A total of 4 independent experiments with different HUVEC cultures were performed.

Since it has been demonstrated that HO-1 is able to inhibit NFκB p65 phosphorylation at Ser276 [34] and because HO-1 was strongly induced by NOD, we sought to assess the contribution of HO-1 on NOD mediated inhibition of NFκB. To this end, we employed an siRNA approach to either knock-down NF-E2 related factor-2 (Nrf-2) expression, a transcription factor that drives the expression of HO-1, or by knock-down of HO-1 expression directly (figure 4). As described by the supplier, neither Nrf-2 nor HO-1 siRNA completely blocked HO-1 expression, yet, even though HO-1 expression was significantly diminished by these siRNAs the inhibitory effect of NOD on VCAM-1 expression was not affected (figure 4). To formerly exclude a role for HO-1 in inhibition of NFκB by NOD, we used a second approach by blocking *de novo* protein synthesis. HUVECs were either pre-incubated with cyclohexamide (CyHx) for 2 hrs or left untreated and subsequently stimulated for 8 hrs with TNF-α alone or in combination with NOD in the continued presence of CyHx. The 8 hrs time period of stimulation was chosen on the basis of CyHx associated cell toxicity that usually occurred after 12 hrs of CyHx treatment. In this experimental setting protein synthesis was effectively blocked by CyHx since induction of VCAM-1 by TNF-α alone or induction of HO-1 by NOD was not observed in the presence of CyHx (figure 5A). Similar as shown in figure 3A, activation of NFκB was evident after 8 hrs of stimulation with TNF-α alone, while it was strongly diminished in combination with NOD (figure 5B). In CyHx pre-treated HUVECs NFκB activation was less pronounced after TNF-α stimulation, yet inhibition was still observed in the combination of TNF-α+NOD (figure 5B).

### Structural Requirements for Inhibition of VCAM-1 by NOD

**Figure 6:**
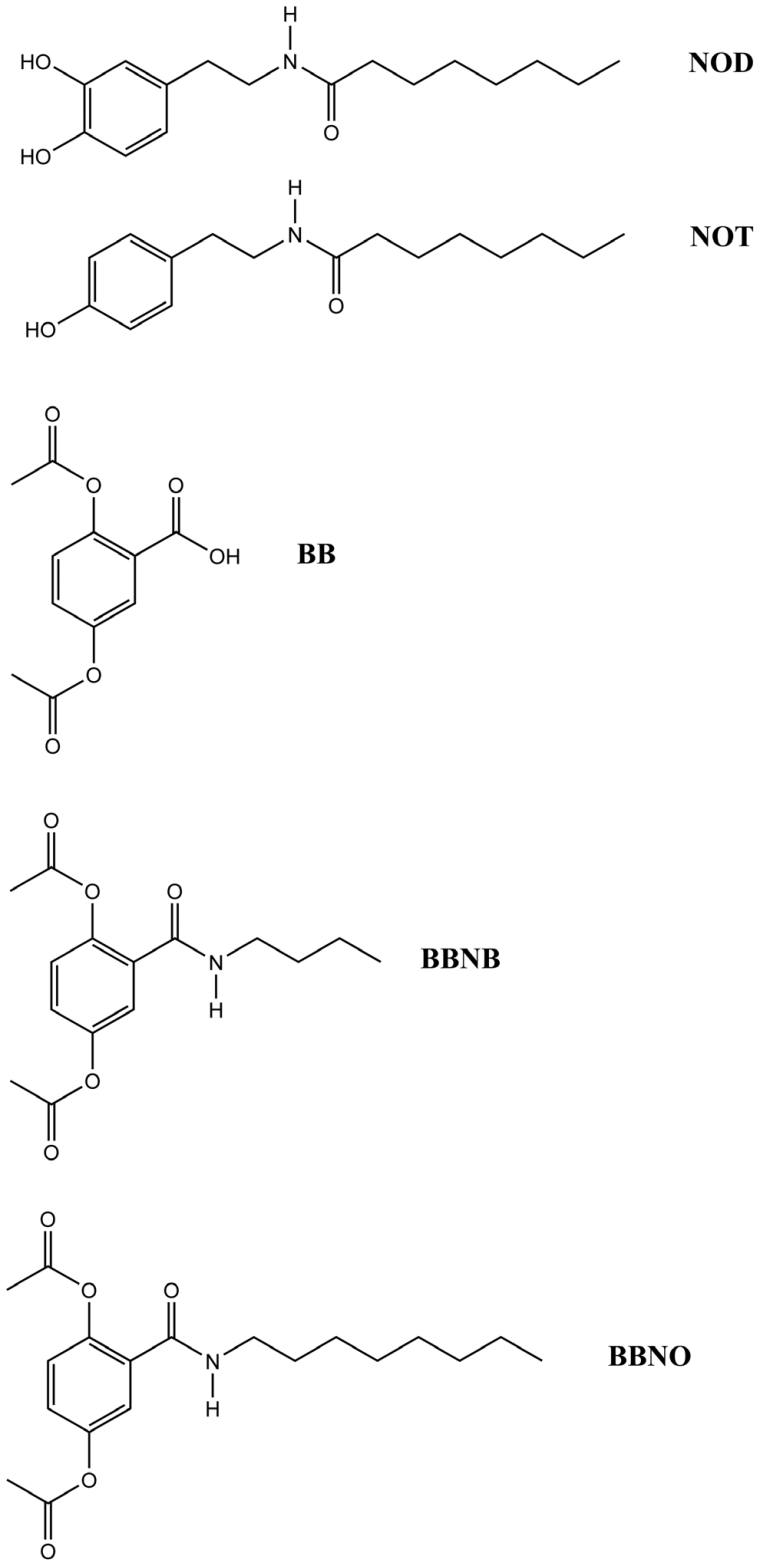
Structure of the compounds used in this study.

To assess the structural entities within NOD that are responsible for its anti-inflammatory effect, we synthesized structurally related compounds that differ in their redox activity or in their hydrophobicity (figure 6). To this end, dopamine or tyramine were covalently bound to octanoic acid at the amine side chain, yielding N-octanoyl dopamine (NOD) or N-octanoyl-tyramine (NOT). These compounds differ in redox activity, while the hydrophobicity of both is not significantly different as calculated by the engine at www.molinspiration.com (3.7 vs. 4.0). In addition we synthesized compounds by sequential modification of the redox active 2,5-dihydroxy-benzoic acid (genestic acid). The dihydroxy moieties were first acetylated resulting in 2,5-bisacetoxy-benzoic acid (BB). This prevents oxidation of the compound, unless redox activity is restored through the action of intra-cellular esterase activity, and facilitates cellular uptake by reducing polarity. In a second step the 2,5-bisacetoxy-benzoic acid was reacted at the free carboxy group with either n-butylamine or n-octylamine, resulting in 2,5-bisacetoxybenzoyl-N-butylamide (BBNB) and 2,5-bisacetoxybenzoyl-N-octanoylamide (BBNO) respectively. These compounds differ in hydrophobicity compared to the parent compound BB, while the redox activity is similar provided that the dihydroxy moiety is restored by intra-cellular esterase activity.

**Figure 7:**
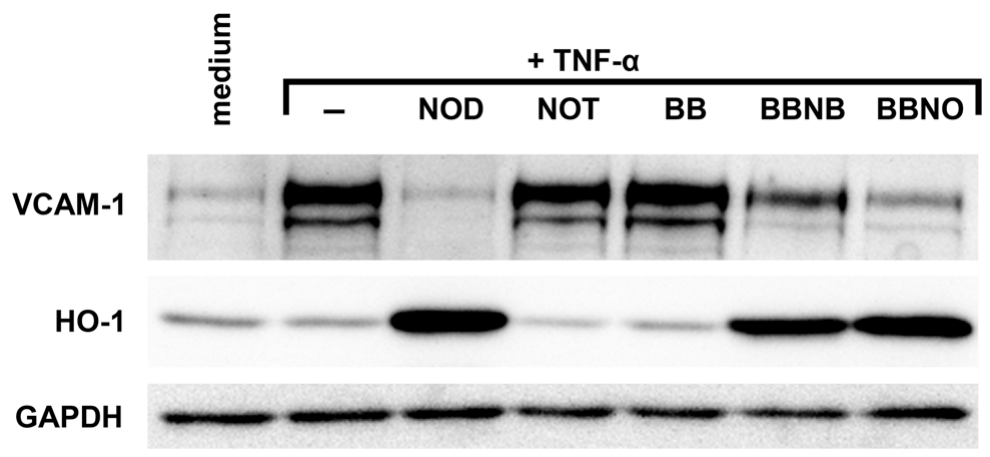
A redox moiety and sufficient hydrophobicity are required for NOD mediated inhibition of VCAM-1 expression and for induction of HO-1. HUVECs were stimulated with 10 ng/ml of TNF-α alone (−) or stimulated with TNF-α in the presence of the different compounds shown in figure 6. Cells that were left untreated (medium) served as control. The expression of VCAM-1 and HO-1 was assessed by western blotting. GAPDH was used as loading control. The results of a representative experiment are shown. A total of 3 independent experiments with different HUVEC cultures were performed.

All compounds were tested for their ability to inhibit TNF-α mediated VCAM-1 expression (figure 7). In contrast to NOD NOT was not able to inhibit VCAM-1 expression indicating that a redox active moiety might be essential for VCAM-1 inhibition. In addition, VCAM-1 inhibition only occurred if the free 2,5-bisacetoxy-benzoic acid was linked to butylamine or octylamine indicating that the inhibition requires sufficient hydrophobicity of the compound. All compounds that were able to inhibit VCAM-1 expression also induced the expression of HO-1.

## Discussion

In the present study we assessed the anti-inflammatory potential of NOD and sought to elucidate the underlying molecular mechanism by which this was mediated. The main findings of this study are the following. Firstly, NOD down-regulates a wide range of κB regulated pro-inflammatory mediators, e.g. chemokines and adhesion molecules, yet not all κB regulated genes were affected by NOD. Down-regulation of inflammatory mediators had functional consequences for the adherence of PBMCs to endothelial cells and was associated with the inhibition of NFκB. Secondly, inhibition of NFκB occurred independently of IκBα degradation and was reflected by an overall decrease in p65 expression and a decreased phosphorylation of p65 Ser276. Thirdly, *de novo* protein synthesis was not required for inhibition of NFκB, hence excluding that up-regulation of HO-1 was involved in the anti-inflammatory properties of NOD. In line with this, it was found that in HO-1 siRNA transfected cells NOD mediated inhibition of VCAM-1 expression was not impaired. Finally, we provide evidence that redox activity and hydrophobicity are important molecular entities that are required for the anti-inflammatory properties of NOD.

Owing to its ability of simultaneously activating multiple signalling pathways, TNF-α regulates a plethora of biological responses in cells, e.g. cell death, proliferation, differentiation and inflammation [35], [36]. This is essentially mediated through the formation of two signalling complexes, mediating NFκB activation and initiating apoptosis respectively [37], [38]. Cross-talk between both platforms occurs via NFκB dependent transcription of anti-apoptotic genes. Inhibition of NFκB therefore instantaneously results in cell death [38]. Interestingly, our data seem to be in contradiction with this notion, as apoptosis was not noticed when HUVECs were simultaneously stimulated with TNF-α and NOD, despite the fact that NFκB was clearly inhibited under this condition. Because degradation of IκBα was not influenced by NOD and not all κB regulated gene transcription was equally affected, our data suggest that NOD does not completely prevent activation of NFκB. Limited activation of NFκB that occurs in the presence of TNF-α and NOD might still be sufficient to drive the transcription of the anti-apoptotic proteins Bcl-xl, c-FLIP and XIAP, which is in line with the observation that Affymetrix analysis did not reveal statistical differences in their expression.

Transcription factor-selective and signal-specific cofactor recruitment is important to ensure restricted gene transcription despite redundancy in signalling pathways http://www.jbc.org/cgi/ijlink?linkType=ABST&journalCode=mcb&resid=19/1/855 [39]–[41]. For many NFκB regulated genes, accessibility of κB sites in promoter regions requires DNA uncoiling, a process which is regulated by histone acetyltransferase (HAT) activity [42], [43]. Evidence has culminated in recent years demonstrating that HAT activity is provided to p50/RelA NFκB dimers by the recruitment of CBP/p300. This is essential for transcription of a number of inflammatory genes [44], [45], particularly for genes in which the κB sites are not directly accessible [43]. Although our study did not address if CBP/p300 recruitment is impaired by NOD, we did observe that phosphorylation of RelA (p65) at Ser276 was inhibited by NOD. In keeping with the essential role of p65 Ser276 phosphorylation for recruitment of CBP/p300 [32], [33], it is likely that the selective inhibitory effect of NOD on the transcription of pro-inflammatory genes might be related to the lack of DNA uncoiling required for transcription of these genes, leaving κB regulated genes with direct accessibility unaffected.

Apart from the lack of p65 Ser276 phosphorylation, we also observed an overall decrease in cellular p65 expression in TNF-α plus NOD stimulated cells. The mechanism that underlies this observation is still elusive. In addition to IκBα mediated nuclear export of NFκB, proteasomal degradation of DNA-bound p65 regulates NFκB dependent gene expression [46], [47]. Hence, an increased proteasomal degradation of p65 might account for an overall diminished p65 expression in TNF-α plus NOD stimulated cells. Yet, it should be emphasized that NOD down-regulates several genes involved in ubiquitination and proteasomal degradation, including proteasomal subunits, ubiquitin ligases and ubiquitin like modifiers (table 2). Amongst these genes, the ubiquitin like modifier UBD appeared to be the one that was the most affected by NOD. Recent studies have demonstrated that UBD expression is important for appropriate NFκB activation [48], [49]. In renal tubular epithelial cells derived from UBD ^−/−^ mice TNF-α induced NFκB activation is abrogated as the result of an altered proteasomal subunit expression [48], [49]. Although our data demonstrate a decreased expression for both UBD and proteasomal subunits (LMP2 (PSMB9), LMP7 (PSMB8), LMP10 (PSMB10)), our results do not allow the conclusion that an altered proteasomal subunit expression, as observed in cells that were stimulated in the presence of NOD, is causally related to inhibition of NFκB. Because transcription of UBD and proteasomal subunits is also regulated by NFκB [50], [51], it is more likely that the expression of genes belonging to the ubiquitin proteasomal system is modulated by NOD through an inadequate NFκB activation.

NFκB activation has a dual and opposite dependence on oxidative events, because its translocation is favoured by an oxidative milieu in the cytosol while binding to DNA requires a reductive environment in the nucleus [52]–[54]. Therefore the finding that the anti-inflammatory properties of NOD rely on its redox activity was not surprising and compatible with published studies on polyphenols [55], [56]. Our data are also in line with a previously published study in which it was shown that catechols in caffeic acid phenethyl ester (CAPE) selectively inhibit NFκB target genes [17].

Activation of the Keap-1/Nrf-2 pathway determines the ability of multicellular organisms to adapt to conditions of stress caused by oxidants and electrophiles via induction of proteins with versatile cyto-protective functions such as HO-1. Para- and ortho-dihydroxybenzene derivatives (catechols and hydroquinones) were among the first identified small-molecule inducers of this pathway. Oxidation of these hydroquinones to their corresponding electrophilic quinones is a requisite step for the activation of the Keap-1/Nrf-2 pathway. In line with this, it was found that HO-1 was induced by the catechol NOD and that this was partly dependent on Nrf-2. Also the 2,5-bisacetoxybenzoyl-N-butylamide (BBNB) and 2,5-bisacetoxybenzoyl-N-octanoylamide (BBNO) derivatives, which by virtue of intra-cellular esterase activity are converted to para-hydroquinones, strongly increased HO-1 expression. In contrast, the free 2,5-bisacetoxy-benzoic acid (BB) did not induce HO-1 expression, which might be explained by its lower hydrophobicity and thereby its inefficient cellular uptake. Therefore, apart from its redox activity a sufficient degree of hydrophobicity seems to be important for the anti-inflammatory effect of NOD. This is also supported by our previous finding that inhibition of VCAM-1 expression by dopamine is only partial and occurs at much higher concentrations (300 µM) [22] compared to the present finding on the more hydrophobic NOD.

We are aware of the studies from Soares et al. [57] and Seldon et al. [34] showing a pivotal role for HO-1 in down-regulation of VCAM-1 expression. According to their data HO-1 down-regulates the inflammatory phenotype of endothelial cells by reducing intracellular nonprotein-bound iron [34]. Accordingly, reduction of the labile iron pool results in hypophosphorylation of RelA Ser276. Although we can exclude that the inhibitory effect of NOD on NFκB is mediated via upregulation of HO-1, a reduction of the labile iron pool as cause for RelA Ser276 hypophosphorylation might still be valid. Microorganisms circumvent low iron availability by secreting siderophores that complex ferric iron with high affinity [58]. The chelating functionalities of siderophores include catecholates, hydroxamates, and α-hydroxycarboxylates [59]. Hence, both NOD (catecholate) and the from genestic acid (α-hydroxycarboxylates) derived BB compounds have iron chelating properties. In contrast to these compounds NOT has no iron chelating properties and is not able to inhibit VCAM-1 expression. It remains to be assessed if the importance of the redox moiety of NOD for its inhibitory effect is related to impairment of the redox milieu within intracellular compartments, its iron chelating functionality or both.

In conclusion our data demonstrate that NOD has a potent inhibitory effect on TNF-α mediated inflammatory processes. This occurs, most likely through its action on post-translational modification of NFκB. Inhibition seems to be selective, affecting only the expression of a subset of κB regulated genes. Although redox modulation of chromatin remodeling may account for selectivity in this regard, the role of iron in RelA Ser276 phosphorylation, its role in chromatin remodelling and if the effect of NOD is only restricted to the TNF-α signalling cascade need to be addressed in further studies.

## References

[1] Oppenheimer-Marks N, Davis LS, Bogue DT, Ramberg J, Lipsky PE (1991) Differential utilization of ICAM-1 and VCAM-1 during the adhesion and transendothelial migration of human T lymphocytes. J Immunol 147: 2913–2921. 1717579

[2] Vestweber D, Winderlich M, Cagna G, Nottebaum AF (2009) Cell adhesion dynamics at endothelial junctions: VE-cadherin as a major player. Trends Cell Biol 19: 8–15. 19010680 10.1016/j.tcb.2008.10.001

[3] Muller WA (2003) Leukocyte-endothelial-cell interactions in leukocyte transmigration and the inflammatory response. Trends Immunol 24: 327–334. 12810109 10.1016/s1471-4906(03)00117-0

[4] Vestweber D (2007) Adhesion and signaling molecules controlling the transmigration of leukocytes through endothelium. Immunol Rev 218: 178–196. 17624953 10.1111/j.1600-065X.2007.00533.x

[5] Carman CV, Springer TA (2008) Trans-cellular migration: cell-cell contacts get intimate. Curr Opin Cell Biol 20: 533–540. 18595683 10.1016/j.ceb.2008.05.007 PMC2811962

[6] Ghosh S, Hayden MS (2008) New regulators of NF-kappaB in inflammation. Nat Rev Immunol 8: 837–848. 18927578 10.1038/nri2423

[7] Ferreiro DU, Komives EA (2010) Molecular mechanisms of system control of NF-kappaB signaling by IkappaBalpha. Biochemistry 49: 1560–1567. 20055496 10.1021/bi901948j PMC2865148

[8] Yamamoto Y, Gaynor RB (2001) Therapeutic potential of inhibition of the NF-kappaB pathway in the treatment of inflammation and cancer. The Journal of clinical investigation 107: 135–142. 11160126 10.1172/JCI11914 PMC199180

[9] Hommelberg PP, Langen RC, Schols AM, Mensink RP, Plat J (2010) Inflammatory signaling in skeletal muscle insulin resistance: green signal for nutritional intervention? Curr Opin Clin Nutr Metab Care 13: 647–655. 20842028 10.1097/MCO.0b013e32833f1acd

[10] Nair HB, Sung B, Yadav VR, Kannappan R, Chaturvedi MM, et al (2010) Delivery of antiinflammatory nutraceuticals by nanoparticles for the prevention and treatment of cancer. Biochem Pharmacol 80: 1833–1843. 20654584 10.1016/j.bcp.2010.07.021 PMC2974020

[11] Sundar IK, Chung S, Hwang JW, Lapek JD Jr, Bulger M, et al (2012) Mitogen- and stress-activated kinase 1 (MSK1) regulates cigarette smoke-induced histone modifications on NF-kappaB-dependent genes. PLoS One 7: e31378. 22312446 10.1371/journal.pone.0031378 PMC3270039

[12] Anand P, Sung B, Kunnumakkara AB, Rajasekharan KN, Aggarwal BB (2011) Suppression of pro-inflammatory and proliferative pathways by diferuloylmethane (curcumin) and its analogues dibenzoylmethane, dibenzoylpropane, and dibenzylideneacetone: role of Michael acceptors and Michael donors. Biochem Pharmacol 82: 1901–1909. 21924245 10.1016/j.bcp.2011.09.001 PMC3216474

[13] Dileep KV, Tintu I, Mandal PK, Karthe P, Haridas M, et al (2012) Binding to PLA2 may contribute to the anti-inflammatory activity of catechol. Chem Biol Drug Des 79: 143–147. 21995306 10.1111/j.1747-0285.2011.01258.x

[14] Dinkova-Kostova AT, Wang XJ (2011) Induction of the Keap1/Nrf2/ARE pathway by oxidizable diphenols. Chem Biol Interact 192: 101–106. 20846517 10.1016/j.cbi.2010.09.010

[15] Kim AN, Jeon WK, Lee JJ, Kim BC (2010) Up-regulation of heme oxygenase-1 expression through CaMKII-ERK1/2-Nrf2 signaling mediates the anti-inflammatory effect of bisdemethoxycurcumin in LPS-stimulated macrophages. Free Radic Biol Med 49: 323–331. 20430097 10.1016/j.freeradbiomed.2010.04.015

[16] Lee Y, Shin DH, Kim JH, Hong S, Choi D, et al (2010) Caffeic acid phenethyl ester-mediated Nrf2 activation and IkappaB kinase inhibition are involved in NFkappaB inhibitory effect: structural analysis for NFkappaB inhibition. Eur J Pharmacol 643: 21–28. 20599928 10.1016/j.ejphar.2010.06.016

[17] Mapesa JO, Waldschmitt N, Schmoeller I, Blume C, Hofmann T, et al (2011) Catechols in caffeic acid phenethyl ester are essential for inhibition of TNF-mediated IP-10 expression through NF-kappaB-dependent but HO-1- and p38-independent mechanisms in mouse intestinal epithelial cells. Mol Nutr Food Res 55: 1850–1861. 22038897 10.1002/mnfr.201100105

[18] Ader R, Cohen N, Felten D (1995) Psychoneuroimmunology: interactions between the nervous system and the immune system. Lancet 345: 99–103. 7815892 10.1016/s0140-6736(95)90066-7

[19] Beck G, Brinkkoetter P, Hanusch C, Schulte J, van Ackern K, et al (2004) Clinical review: immunomodulatory effects of dopamine in general inflammation. Crit Care 8: 485–491. 15566620 10.1186/cc2879 PMC1065039

[20] Berger SP, Hunger M, Yard BA, Schnuelle P, Van Der Woude FJ (2000) Dopamine induces the expression of heme oxygenase-1 by human endothelial cells in vitro. Kidney international 58: 2314–2319. 11115065 10.1046/j.1523-1755.2000.00415.x

[21] Raddatz A, Kubulus D, Winning J, Bauer I, Pradarutti S, et al (2006) Dobutamine improves liver function after hemorrhagic shock through induction of heme oxygenase-1. Am J Respir Crit Care Med 174: 198–207. 16627864 10.1164/rccm.200508-1221OC

[22] Beck GC, Oberacker R, Kapper S, von Zabern D, Schulte J, et al (2001) Modulation of chemokine production in lung microvascular endothelial cells by dopamine is mediated via an oxidative mechanism. Am J Respir Cell Mol Biol 25: 636–643. 11713107 10.1165/ajrcmb.25.5.4544

[23] Kapper S, Beck G, Riedel S, Prem K, Haak M, et al (2002) Modulation of chemokine production and expression of adhesion molecules in renal tubular epithelial and endothelial cells by catecholamines. Transplantation 74: 253–260. 12151739 10.1097/00007890-200207270-00017

[24] Hoeger S, Gottmann U, Liu Z, Schnuelle P, Birck R, et al (2007) Dopamine treatment in brain-dead rats mediates anti-inflammatory effects: the role of hemodynamic stabilization and D-receptor stimulation. Transpl Int 20: 790–799. 17596177 10.1111/j.1432-2277.2007.00510.x

[25] Gottmann U, Brinkkoetter PT, Bechtler M, Hoeger S, Karle C, et al (2006) Effect of pre-treatment with catecholamines on cold preservation and ischemia/reperfusion-injury in rats. Kidney international 70: 321–328. 16760913 10.1038/sj.ki.5001501

[26] Losel RM, Schnetzke U, Brinkkoetter PT, Song H, Beck G, et al (2010) N-octanoyl dopamine, a non-hemodyanic dopamine derivative, for cell protection during hypothermic organ preservation. PLoS One 5: e9713. 20300525 10.1371/journal.pone.0009713 PMC2838791

[27] Tsagogiorgas C, Wedel J, Hottenrott M, Schneider MO, Binzen U, et al (2012) N-octanoyl-dopamine is an agonist at the capsaicin receptor TRPV1 and mitigates is chemia-induced acute kidney injury in rat. PLoS One 7: e43525. 22916273 10.1371/journal.pone.0043525 PMC3423369

[28] Song H, Bergstrasser C, Rafat N, Hoger S, Schmidt M, et al (2009) The carbon monoxide releasing molecule (CORM-3) inhibits expression of vascular cell adhesion molecule-1 and E-selectin independently of haem oxygenase-1 expression. Br J Pharmacol 157: 769–780. 19422386 10.1111/j.1476-5381.2009.00215.x PMC2721262

[29] Beck GC, Rafat N, Brinkkoetter P, Hanusch C, Schulte J, et al (2006) Heterogeneity in lipopolysaccharide responsiveness of endothelial cells identified by gene expression profiling: role of transcription factors. Clin Exp Immunol 143: 523–533. 16487252 10.1111/j.1365-2249.2006.03005.x PMC1809605

[30] Anrather J, Csizmadia V, Soares MP, Winkler H (1999) Regulation of NF-kappaB RelA phosphorylation and transcriptional activity by p21(ras) and protein kinase Czeta in primary endothelial cells. J Biol Chem 274: 13594–13603. 10224130 10.1074/jbc.274.19.13594

[31] Brouard S, Berberat PO, Tobiasch E, Seldon MP, Bach FH, et al (2002) Heme oxygenase-1-derived carbon monoxide requires the activation of transcription factor NF-kappa B to protect endothelial cells from tumor necrosis factor-alpha-mediated apoptosis. J Biol Chem 277: 17950–17961. 11880364 10.1074/jbc.M108317200

[32] Vermeulen L, De Wilde G, Van Damme P, Vanden Berghe W, Haegeman G (2003) Transcriptional activation of the NF-kappaB p65 subunit by mitogen- and stress-activated protein kinase-1 (MSK1). The EMBO journal 22: 1313–1324. 12628924 10.1093/emboj/cdg139 PMC151081

[33] Reber L, Vermeulen L, Haegeman G, Frossard N (2009) Ser276 phosphorylation of NF-kB p65 by MSK1 controls SCF expression in inflammation. PLoS One 4: e4393. 19197368 10.1371/journal.pone.0004393 PMC2632887

[34] Seldon MP, Silva G, Pejanovic N, Larsen R, Gregoire IP, et al (2007) Heme oxygenase-1 inhibits the expression of adhesion molecules associated with endothelial cell activation via inhibition of NF-kappaB RelA phosphorylation at serine 276. J Immunol 179: 7840–7851. 18025230 10.4049/jimmunol.179.11.7840

[35] Simeonova PP, Gallucci RM, Hulderman T, Wilson R, Kommineni C, et al (2001) The role of tumor necrosis factor-alpha in liver toxicity, inflammation, and fibrosis induced by carbon tetrachloride. Toxicol Appl Pharmacol 177: 112–120. 11740910 10.1006/taap.2001.9304

[36] Wajant H, Pfizenmaier K, Scheurich P (2003) Tumor necrosis factor signaling. Cell Death Differ 10: 45–65. 12655295 10.1038/sj.cdd.4401189

[37] Hsu H, Xiong J, Goeddel DV (1995) The TNF receptor 1-associated protein TRADD signals cell death and NF-kappa B activation. Cell 81: 495–504. 7758105 10.1016/0092-8674(95)90070-5

[38] Micheau O, Tschopp J (2003) Induction of TNF receptor I-mediated apoptosis via two sequential signaling complexes. Cell 114: 181–190. 12887920 10.1016/s0092-8674(03)00521-x

[39] Ikeda K, Steger DJ, Eberharter A, Workman JL (1999) Activation domain-specific and general transcription stimulation by native histone acetyltransferase complexes. Mol Cell Biol 19: 855–863. 9858608 10.1128/mcb.19.1.855 PMC83942

[40] Korzus E, Torchia J, Rose DW, Xu L, Kurokawa R, et al (1998) Transcription factor-specific requirements for coactivators and their acetyltransferase functions. Science 279: 703–707. 9445475 10.1126/science.279.5351.703

[41] Perissi V, Dasen JS, Kurokawa R, Wang Z, Korzus E, et al (1999) Factor-specific modulation of CREB-binding protein acetyltransferase activity. Proceedings of the National Academy of Sciences of the United States of America 96: 3652–3657. 10097092 10.1073/pnas.96.7.3652 PMC22349

[42] Feng D, Sangster-Guity N, Stone R, Korczeniewska J, Mancl ME, et al (2010) Differential requirement of histone acetylase and deacetylase activities for IRF5-mediated proinflammatory cytokine expression. J Immunol 185: 6003–6012. 20935208 10.4049/jimmunol.1000482 PMC3233222

[43] Natoli G (2009) Control of NF-kappaB-dependent transcriptional responses by chromatin organization. Cold Spring Harb Perspect Biol 1: a000224. 20066094 10.1101/cshperspect.a000224 PMC2773620

[44] Okazaki T, Sakon S, Sasazuki T, Sakurai H, Doi T, et al (2003) Phosphorylation of serine 276 is essential for p65 NF-kappaB subunit-dependent cellular responses. Biochemical and biophysical research communications 300: 807–812. 12559944 10.1016/s0006-291x(02)02932-7

[45] Zhong H, Voll RE, Ghosh S (1998) Phosphorylation of NF-kappa B p65 by PKA stimulates transcriptional activity by promoting a novel bivalent interaction with the coactivator CBP/p300. Mol Cell 1: 661–671. 9660950 10.1016/s1097-2765(00)80066-0

[46] Ryo A, Suizu F, Yoshida Y, Perrem K, Liou YC, et al (2003) Regulation of NF-kappaB signaling by Pin1-dependent prolyl isomerization and ubiquitin-mediated proteolysis of p65/RelA. Mol Cell 12: 1413–1426. 14690596 10.1016/s1097-2765(03)00490-8

[47] Saccani S, Marazzi I, Beg AA, Natoli G (2004) Degradation of promoter-bound p65/RelA is essential for the prompt termination of the nuclear factor kappaB response. J Exp Med 200: 107–113. 15226358 10.1084/jem.20040196 PMC2213320

[48] Dev S, Mizuguchi H, Das AK, Baba Y, Fukui H (2011) Transcriptional microarray analysis reveals suppression of histamine signaling by Kujin alleviates allergic symptoms through down-regulation of FAT10 expression. Int Immunopharmacol. 10.1016/j.intimp.2011.05.004 21601015

[49] Gong P, Canaan A, Wang B, Leventhal J, Snyder A, et al (2010) The ubiquitin-like protein FAT10 mediates NF-kappaB activation. J Am Soc Nephrol 21: 316–326. 19959714 10.1681/ASN.2009050479 PMC2834541

[50] Canaan A, Yu X, Booth CJ, Lian J, Lazar I, et al (2006) FAT10/diubiquitin-like protein-deficient mice exhibit minimal phenotypic differences. Mol Cell Biol 26: 5180–5189. 16782901 10.1128/MCB.00966-05 PMC1489174

[51] Wright KL, White LC, Kelly A, Beck S, Trowsdale J, et al (1995) Coordinate regulation of the human TAP1 and LMP2 genes from a shared bidirectional promoter. J Exp Med 181: 1459–1471. 7699330 10.1084/jem.181.4.1459 PMC2191963

[52] Jornot L, Petersen H, Junod AF (1997) Modulation of the DNA binding activity of transcription factors CREP, NFkappaB and HSF by H2O2 and TNF alpha. Differences between in vivo and in vitro effects. FEBS Lett 416: 381–386. 9373190 10.1016/s0014-5793(97)01244-1

[53] Staal FJ, Roederer M, Herzenberg LA (1990) Intracellular thiols regulate activation of nuclear factor kappa B and transcription of human immunodeficiency virus. Proceedings of the National Academy of Sciences of the United States of America 87: 9943–9947. 2263644 10.1073/pnas.87.24.9943 PMC55290

[54] Toledano MB, Leonard WJ (1991) Modulation of transcription factor NF-kappa B binding activity by oxidation-reduction in vitro. Proceedings of the National Academy of Sciences of the United States of America 88: 4328–4332. 1903539 10.1073/pnas.88.10.4328 PMC51652

[55] Rahman I (2008) Dietary polyphenols mediated regulation of oxidative stress and chromatin remodeling in inflammation. Nutr Rev 66 Suppl 1S42–45. 18673489 10.1111/j.1753-4887.2008.00067.x PMC2556856

[56] Rahman I, Marwick J, Kirkham P (2004) Redox modulation of chromatin remodeling: impact on histone acetylation and deacetylation, NF-kappaB and pro-inflammatory gene expression. Biochem Pharmacol 68: 1255–1267. 15313424 10.1016/j.bcp.2004.05.042

[57] Soares MP, Seldon MP, Gregoire IP, Vassilevskaia T, Berberat PO, et al (2004) Heme oxygenase-1 modulates the expression of adhesion molecules associated with endothelial cell activation. J Immunol 172: 3553–3563. 15004156 10.4049/jimmunol.172.6.3553

[58] Neilands JB (1995) Siderophores: structure and function of microbial iron transport compounds. J Biol Chem 270: 26723–26726. 7592901 10.1074/jbc.270.45.26723

[59] Correnti C, Strong RK (2012) Mammalian siderophores, siderophore-binding lipocalins, and the labile iron pool. J Biol Chem 287: 13524–13531. 22389496 10.1074/jbc.R111.311829 PMC3340207

